# Treatment plan quality during online adaptive re-planning

**DOI:** 10.1186/s13014-020-01641-0

**Published:** 2020-08-21

**Authors:** Janita E. van Timmeren, Madalyne Chamberlain, Jérôme Krayenbuehl, Lotte Wilke, Stefanie Ehrbar, Marta Bogowicz, Callum Hartley, Mariangela Zamburlini, Nicolaus Andratschke, Helena Garcia Schüler, Matea Pavic, Panagiotis Balermpas, Chaehee Ryu, Matthias Guckenberger, Stephanie Tanadini-Lang

**Affiliations:** Department of Radiation Oncology, University Hospital Zürich and University of Zürich, Rämistrasse 100, 8091 Zürich, Switzerland

**Keywords:** Radiotherapy, MR-linac, Online-adaptive radiation therapy, MR-guided, MRgRT, Online, Adaptive, Planning, SBRT

## Abstract

**Background:**

Online adaptive radiotherapy is intended to prevent plan degradation caused by inter-fractional tumor volume and shape changes, but time limitations make online re-planning challenging. The aim of this study was to compare the quality of online-adapted plans to their respective reference treatment plans.

**Methods:**

Fifty-two patients treated on a ViewRay MRIdian Linac were included in this retrospective study. In total 238 online-adapted plans were analyzed, which were optimized with either changing of the segment weights (*n* = 85) or full re-optimization (*n* = 153). Five different treatment sites were evaluated: prostate, abdomen, liver, lung and pelvis. Dosimetric parameters of gross tumor volume (GTV), planning target volume (PTV), 2 cm ring around the PTV and organs at risk (OARs) were considered. The Wilcoxon signed-rank test was used to assess differences between online-adapted and reference treatment plans, *p* < 0.05 was considered significant.

**Results:**

The average duration of the online adaptation, consisting of contour editing, plan optimization and quality assurance (QA), was 24 ± 6 min. The GTV was slightly larger (average ± SD: 1.9% ± 9.0%) in the adapted plans than in the reference plans (*p* < 0.001). GTV-D_95%_ exhibited no significant changes when considering all plans, but GTV-D_2%_ increased by 0.40% ± 1.5% on average (*p* < 0.001). There was a very small yet significant decrease in GTV-coverage for the abdomen plans. The ring D_mean_ increased on average by 1.0% ± 3.6% considering all plans (*p* < 0.001). There was a significant reduction of the dose to the rectum of 4.7% ± 16% on average (*p* < 0.001) for prostate plans.

**Conclusions:**

Dosimetric quality of online-adapted plans was comparable to reference treatment plans and OAR dose was either comparable or decreased, depending on treatment site. However, dose spillage was slightly increased.

## Background

Developments in image-guided radiotherapy (IGRT) contribute to more precise and accurate radiation treatment delivery. Imaging patients immediately prior to or during treatment improves patient set-up and makes it possible to perform real-time corrections, which prevent plan degradations caused by tumor progression and anatomical changes [1]. These online adaptations allow for reduced safety margins in the planning phase, increased tumor dose, and better sparing of organs at risk (OARs) [2]. This could eventually lead to improved tumor control and reduced toxicity.

Modern linear accelerators (linacs) are often equipped with a cone-beam computed tomography (CBCT) system, which allows for two-, three and four-dimensional patient positioning verification. Although CBCT images are able to detect anatomical changes and trigger adaptive re-planning, IGRT is limited by the CBCT’s increased noise level and low soft-tissue contrast, especially in the abdomen and pelvic regions, when compared to conventional CT. Moreover, CBCT acquisitions deposit additional dose outside the treatment volume.

The combination of magnetic resonance imaging (MRI) and linac (MR-linac) makes it possible to use imaging with superior soft tissue contrast during the course of radiation treatment and in the treatment position, without adding imaging dose to the patient [3–5]. These properties make an MR-linac suitable for MR-guided adaptive radiotherapy (MRgART), which includes MR-guided radiation delivery as well as online treatment plan adaptations to match daily anatomy [6]. Previous studies have shown that it was possible to achieve a plan quality with an MR-linac that was comparable to that of conventional linacs [7–9]. MR-linacs are currently installed in multiple centers worldwide, and several studies have reported on their experience of using MR-linacs for online-adaptive radiotherapy [10–13]. The use of MRgART was previously described for rectum, prostate, abdomen (pancreas and liver), lung, and the pelvic region [7, 11, 12, 14–20]. These studies showed the feasibility of MRgART and the possibility to reduce planning target volume (PTV) margins and limit OAR doses.

In the MR-guided online-adaptive workflow, daily MR images are acquired while the patient is in the treatment position and registered to the reference MRI scan. Thereafter, contours are edited, and the treatment plan is recalculated on the new MRI and re-optimized. Previous studies have reported a duration of around 50 min for the online-adaptive workflow [11, 12, 14, 15]. Since the patients are in the treatment position during this entire procedure, the aim is to perform efficient and fast online adaptations to improve patient comfort and to avoid anatomical changes during this lap of time (i.e. intra-fractional changes). However, despite the limited time available for adapting the treatment plan according to the daily anatomy, it is important that the plan quality remains high. Therefore, in order to detect potential plan quality degradations for patients receiving daily adapted treatments, the aim of this study was to retrospectively compare the (dosimetric) plan quality of all online-adapted treatment plans to their respective reference treatment plans, which were considered optimal.

## Materials and methods

A ViewRay MRIdian Linac (Viewray, Inc., Oakwood Village, Ohio, USA) has been operational at the University Hospital of Zürich since April 2019. The MRIdian is a 0.345 T MRI integrated with a 6 MV flattening filter-free (FFF) linac [21, 22]. The machine is able to deliver a dose rate of 600 cGy/min at 90 cm source-to-axis distance (SAD). It is equipped with a double-stacked double-focused multi-leaf collimator (MLC) with a leaf width of 8.3 mm at the isocentric plane, corresponding to an effective leaf width of 4.15 mm. The MRIdian has an integrated adaptive treatment planning system (TPS) that allows for online adaptation of treatment plans based on an MRI acquired in treatment position.

### Patients

All patients that received daily adaptive treatments on the MRIdian between April 2019 and November 2019, in total 52, were included in this retrospective analysis. This includes patients with the tumor sites lung, prostate, liver, pelvis, pancreas, kidney, adrenal gland, or abdomen (other). The tumor sites pancreas, kidney, adrenal gland, and abdomen (other) were analyzed together as ‘abdomen’. Details about the patients and treatments included in the study are presented in Table 1.

**Table 1:** Overview of the patients included in this study. “n=” Indicates the number of patients. FX = fractions; GTV = Gross Tumor Volume, PTV = Planning Target Volume, OAR = Organ at Risk

Treatment	Number of treatments	Fractionation	Treatment technique	Total number of FX		Optimization method		GTV median [range] (cm^**3**^)	PTV median [range] (cm^**3**^)	OAR
				Planned	Delivered	Full	Weight			
Abdomen	18			84	81	52 (64%)	29 (36%)			
Pancreas	3	5*6.6 Gy (*n* = 2) 5*8.0 Gy (*n* = 1)	SBRT (*n* = 3)	15	15	13	2	16.1 [1.4–53.9]	45.5 [8.7–78.8]	Duodenum (*n* = 1) Bowel (*n* = 1) Stomach (*n* = 1)
Kidney	2^a^	5*6.0 Gy (*n* = 1) 5*8.0 Gy (*n* = 1)	SBRT (*n* = 2)	9	9	5	4	14.1 [2.1–26.0]	37.5 [8.4–66.6]	Bowel (*n* = 1)
Adrenal gland	5	5*6.0 Gy (*n* = 3) 5*7.0 Gy (*n* = 2)	SBRT (*n* = 5)	25	22^d^	10	12	20.7 [11.2–30.2]	59.8 [33.2–105.7]	Duodenum (*n* = 2) Bowel (*n* = 3)
Abdomen, other	8	2*5.0 Gy (*n* = 1) 3*3.0 Gy (*n* = 1) 5*6.0 Gy (*n* = 2) 5*6.6 Gy (*n* = 1) 5*7.0 Gy (*n* = 1) 5*8.0 Gy (*n* = 1) 5*9.0 Gy (*n* = 1)	non-SBRT (*n* = 1) SBRT (*n* = 7)	35	35	24	11	8.7 [2.2–58.9]	27.8 [10.6–92.2]	Duodenum (*n* = 1) Bowel (*n* = 3) Stomach (*n* = 3)
Prostate	13			55	55	32 (58%)	22 (40%)			
Prostate SBRT	8	1*5.0 Gy (*n* = 1) 5*7.0 Gy (*n =* 1) 5*7.25 Gy (*n* = 6)	SBRT (*n* = 8)	36	36	27	9	29.2 [1.9–50.5]	29.2 [11.8–86.2]	Rectum (*n* = 8) Bladder (*n* = 8)
Prostate IMRT	5	3*2.0 Gy (*n* = 3) 4*2.0 Gy (*n* = 1) 6*2.0 Gy (*n* = 1)	non-SBRT (*n* = 5)	19	19	5	13	1.8 [0.77–3.3]	11.3 [5.7–12.1]	Rectum (*n* = 5) Bladder (*n* = 5)
Lung	10^b^	3*10 Gy (*n* = 1) 3*12.5 Gy (*n* = 3) 5*8.0 Gy (*n* = 1) 5*9.0 Gy (*n* = 3) 8*6.0 Gy (*n* = 2)	SBRT (*n* = 10)	48	48	28 (58%)	13 (27%)	4.1 [0.54–67.6]	17.1 [4.6–131.3]	
Liver	9			38	38	31 (82%)	7 (18%)			
Liver SBRT	5^a^	5*6.0 Gy (*n* = 1) 5*9.0 Gy (*n* = 3) 6*5.0 Gy (*n* = 1)	SBRT (*n* = 5)	26	26	21	5	25.5 [6.8–122.2]	67.4 [37.0–292.2]	Bowel (*n* = 1)
Liver IMRT	4	3*3.0 Gy (*n* = 4)	non-SBRT (*n* = 4)	12	12	10	2	20.2 [10.9–27.8]	75.7 [46.8–100.3]	Duodenum (*n* = 4)
Pelvis	5^c^	5*6.5 Gy (*n* = 1) 5*7.0 Gy (*n* = 4)	SBRT (*n* = 5)	25	25	10 (40%)	14 (56%)	4.3 [0.32–5.4]	12.7 [2.5–19.3]	Bowel (*n* = 2)

^a^One patient had two lesions: kidney and liver

^b^One patient had two lung lesions

^c^One patient had two pelvis lesions

^d^Patient continued treatment on conventional linac

### Treatment planning

For treatment planning purposes, an MRI scan was performed with the MR-linac in treatment position. Additionally, a CT scan in the same position and with coils was acquired, usually within a 1 h timeframe, in order to obtain the electron density information required for dose calculation. All MRI scans were acquired using a balanced steady-state free precession (bSSFP) sequence, resulting in T_2_/T_1_-weighted contrast. The in-plane resolution was 1.5 × 1.5 mm, whereas the slice thickness was set to either 1.5 mm for tumors in the pelvic region (scan duration ~ 90 s) or 3 mm for moving tumors (scan duration ~ 23 s), since these patients are imaged in breath-hold. The CT and MRI scans were co-registered, after which relevant structures were contoured on the MRI. During contouring, any functions used for creating targets or planning help structures were saved as ‘rules’; for example, gross tumor volume (GTV) to PTV expansion, or a 2 cm ring around the PTV to control dose conformity. During adaptive re-planning these rules allow the creation of help structures to be performed accurately within seconds.

All patients were treated with step-and-shoot intensity-modulated radiotherapy (IMRT) composed of 9–11 beams, which were often arranged as a ‘pseudo-arc’. The beams were generally spaced evenly around the patient for a central target, whereas the beams are mostly limited to one side of the patient for lateralized targets, depending on exact tumor location. Small changes were made to these beam arrangements to avoid treating through couch edges, or the arms of the patient. For patients with breathing-induced tumor motion the PTV margin around the GTV was set to 7–10 mm in the longitudinal direction and 6–10 mm in the other directions. For tumors in the pelvic region, a margin between 3 and 5 mm was used. Margins were defined according to institutional guidelines, which were based on experience from the conventional linac margins, but slightly adapted for the MR-linac considering increased intra-fractional errors.

The majority of patients were treated using a stereotactic body radiotherapy (SBRT) dose prescription. For the SBRT cases, dose was prescribed to cover 95–99% of the PTV (V_100%_ ≥ 95%). The prescription isodose line was either 65% or 80% of the maximum dose, depending on size of the tumor, proximity of OARs and previous delivered dose (if any). For the 65% isodose prescription, dose maximum (D_0.1cc_) was allowed between 152 and 156%, and the minimum dose to 95% of the GTV (D_95%_) was 135%. For the 80% isodose prescription, D_0.1cc_ was between 123 and 127% and GTV-D_95%_ ≥ 113%. A few patients received a second series boost treatment on the MR-linac, for which a low fraction dose (≤3 Gy) and homogeneous dose prescription was used (referred to as ‘non-SBRT’). For these cases, dose was normalized such that 95% of the PTV received at least 95% of the prescribed dose (PTV-V_95%_ ≥ 95%), and the mean of the PTV (D_mean_) corresponded to 100% of the prescribed dose. Risk-adapted fractionation schemes were used, depending on previous treatments, proximity of OARs, and location of the tumor. OAR constraints are provided in Table 2. Dose calculation was done with a Monte Carlo algorithm that takes into account the magnetic field, and a grid size of 2 mm for SBRT and 3 mm for non-SBRT.

**Table 2:** OAR constraints

Structure	Parameter	SBRT - 3 fractions	SBRT - 5 fractions	SBRT - 8 fractions	SBRT - 10 fractions
		***Tolerance [Gy]***	***Tolerance [Gy]***	***Tolerance [Gy]***	***Tolerance [Gy]***
BrachialPlexus	D1.0cc [Gy]	≤ 24.00	≤ 30.00	≤ 36.00	≤ 40.00
BronchialTree	D1.0cc [Gy]	≤ 32.00	≤ 40.00	≤ 50.00	≤ 54.00
Esophagus	D1.0cc [Gy]	≤ 27.00	≤ 36.00	≤ 43.00	≤ 47.00
Heart	D1.0cc [Gy]	≤ 36.00	≤ 45.00	≤ 55.00	≤ 60.00
SpinalCord_PRV	D1.0cc [Gy]	≤ 19.00	≤ 23.80	≤ 32.00	≤ 35.00
CaudaEquina	D1.0cc [Gy]	≤ 22.00	≤ 27.00	≤ 33.00	≤ 36.00
Trachea	D1.0cc [Gy]	≤ 32.00	≤ 40.00	≤ 50.00	≤ 54.00
Stomach	D1.0cc [Gy]	≤ 21.00	≤ 26.00	≤ 31.00	≤ 33.00
Duodenum	D1.0cc [Gy]	≤ 21.00	≤ 26.00	≤ 31.00	≤ 33.00
Bowel	D1.0cc [Gy]	≤ 21.00	≤ 26.00	≤ 31.00	≤ 33.00
Rectum	D1.0cc [Gy]		≤ 38.00		
Bladder	D1.0cc [Gy]		≤ 40.00		

### MR-guided online-adaptive workflow

Prior to each radiotherapy fraction, a new MRI scan was acquired to capture the anatomy of the day. In order to transfer the segmentations, this MRI scan was matched to the reference MRI using deformable registration. In the first month the MRI was operational, mostly a previously online-adapted MRI instead of the reference MRI was used. But, since we observed that this could cause an accumulation of changes, the standard became to use the reference MRI to transfer the segmentations. The target volume and OAR structures, as well as electron density information were transferred to the new MRI scan. Additionally, inaccuracies in parts of the registrations were overwritten with densities of water, air, lung and bone according to the information of the MRI of the day. A radiation oncologist edited all contours on each slice containing the PTV + 2 cm that required adaptations, including the GTV if necessary, after which the predefined rules were applied. Subsequently, the dose was recalculated on the new MRI scan. This predicted plan was then optimized by means of an automatic optimization of the weights of the static MLC field segments (i.e. weight optimization). Plans were rated acceptable if all OAR constraints were fulfilled while PTV and GTV coverage were as high as possible, allowing for suboptimal coverage while maintaining OAR sparing: for all cases considered in this study, the OARs were higher prioritized than the target. A full new optimization was performed, if this plan did not meet the acceptance criteria. If necessary, the priorities of the objectives were changed in order to reduce the OAR dose or to improve the target coverage. The online-adapted treatment plan was final once both physician and physicist approved the plan, considering the OAR constraints (Table 2). Online quality assurance (QA) consists of a gamma analysis between the TPS dose calculation and an independent Monte-Carlo (MC) dose calculation, and a verification of path length, plan complexity, integral dose and a point-dose calculation using a simple Clarkson algorithm.

**Fig. 1:**
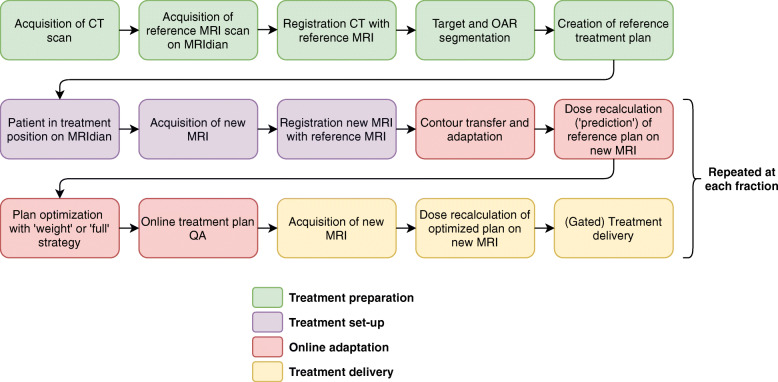
Workflow of treatment using MR-guided online-adaptive radiotherapy on the MRIdian linac

The treatment workflow is summarized in Fig. 1.

### Plan evaluation

The GTV and PTV volumes of the contours in the online-adapted treatment plans were compared with the reference treatment plans. Furthermore, a subgroup analysis was performed to assess whether differences between online-adapted plans and references plans were larger for online-adapted plans that were adapted from a previously online-adapted plan or from the reference plan.

Besides volumes, the following dose-volume histogram (DVH) parameters were evaluated for both GTV and PTV: mean dose (D_mean_), dose to 95% of the volume (D_95%_), dose to 98% of the volume (D_98%_), and dose to 2% of the volume (D_2%_). Additionally, the D_mean_ and dose to 5% of the volume (D_5%_) of the 2 cm ring around the PTV were evaluated, to assess dose conformity and spillage. The dose to 1.0 cc (D_1.0cc_) of the organ in closest proximity with the 2 cm ring was evaluated. For abdomen, liver, and pelvis patients this was either stomach, duodenum or bowel. For prostate patients, the D_1.0cc_ in the rectum as well as in the bladder was evaluated. All described dose parameters were extracted from both, the reference treatment plans and the online-adapted treatment plans, and compared. The number of treatment plans that required either the weight or full optimization strategy was also evaluated. In addition, the differences in dose parameters between both optimization strategies were assessed. Besides a plan-wise comparison, the average values for all online-adapted plans were calculated per patient, in order to be able to identify patients for which all adapted plans had superior or inferior quality compared to the reference plans.

To be clear, all analysis in this study compared the online-adapted treatment plans to the reference treatment plans, which were made prior to treatment and were considered optimal plans.

Besides the dose parameters, the average total time the patients were on the table, as well as the average adaptation time, was calculated for those patients for which this information was recorded. The adaptation time consists of the time required to edit the contours, optimize the treatment plan and online QA, responding to the ‘online adaptation’ steps in the workflow shown in Fig. 1.

### Statistics

Wilcoxon signed-rank test was used to evaluate the differences between individual dose statistics of the online-adapted plans and the reference treatment plans, adopting the null hypothesis that for each metric there are no differences between the two plans. The test was performed using the function *wilcox.test* of the *stats* package in R (version 3.5.0). *P*-values below 0.05 were considered significant.

## Results

At the time this study was conducted, 87 patients were treated on the MRIdian at our center since the installation. In total, 52 patients received daily adaptations and were included in the current analysis. These patients received a total of 55 treatment courses and 247 treatment fractions. For 51/55 treatment courses, the treatment time was recorded at least once during the treatment course. The time was not recorded for two non-SBRT liver patients, one lung patient and one adrenal gland patient. Additionally, for one lung patient the adaptation time was not recorded. The mean ± SD of the average total time the patient (*n* = 51) was on the table was 65 ± 9 min. The mean ± SD of the average adaptation time per patient (*n* = 50) was 24 ± 6 min. For the reference treatment plans there was no time pressure, and a planner typically makes two treatment plans per day.

For three patients, a new reference treatment plan was created during the course of treatment: for one patient the predefined rules were done incorrectly and the plan had to be redone, for one patient the plan was undeliverable since the patient could not be positioned in the required lateral position, and the third patient had problems with holding breath so the beam-on time had to be reduced. Out of all delivered fractions (*n* = 247), for nine fractions the online-adapted plan was not delivered, due to the adaptive QA failing. Therefore, in total 238 online-adapted plans were analyzed in this study. 85/238 plans were optimized using weight optimization, whereas for the remaining 153 plans a full optimization was performed. For 37 out of 238 online-adapted plans, a previous online-adapted plan was used to recalculate the dose on the new MRI, instead of the reference plan. Sixteen out of 37 originated from the first month the MRIdian was operational.

**Fig. 2:**
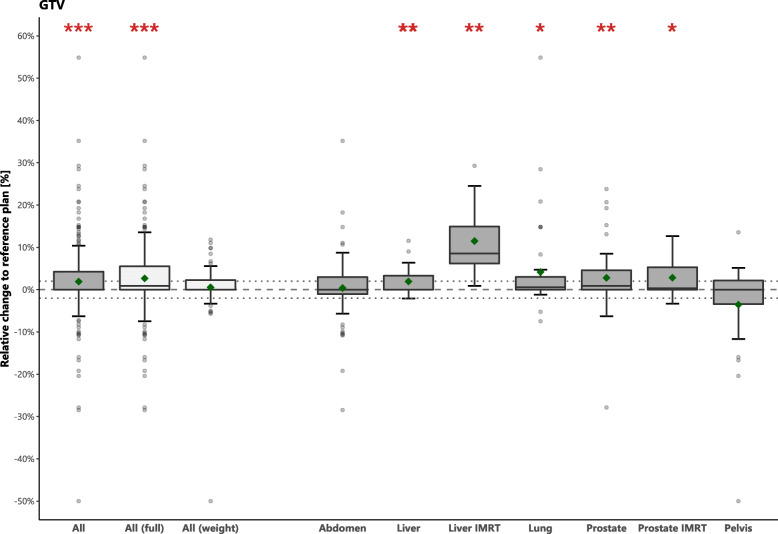
Boxplots indicating the relative volume difference in GTV of the adapted plans compared to the respective reference treatment plans. Diamonds indicate the mean. Dashed lines indicate 0% and dotted lines indicate ±2% differences. Asterisks indicate significance: *p*-value< 0.05 (*), *p*-value< 0.01 (**) and *p*-value< 0.001 (***)

The volume change of the GTV with respect to the reference treatment plans are shown as percentages in Fig. 2, whereas the results for PTV are shown in Figure S1 of [Additional file 1]. For 67/238 (28%) adapted plans, the GTV was equal to the reference treatment plan. Thirty-six of those required a full optimization. For 84% of the adapted plans, the GTV change was ≤1 cm^3^. The GTV and PTV in the adapted treatment plans changed by 1.9 and 2.2% on average, which corresponds to a median [range] of 0.0 cm^3^ [− 5.6–12.0] and 0.1 cm^3^ [− 9.9–23.7], respectively. For 89/238 adapted plans, the GTV increased on average more than 2%. For this group, the majority of plans (67/89, 75.3%) were optimized using full optimization. The GTV changed by less than 2% for the other 149/238 adapted plans, and 86/149 (57.7%) of these plans were optimized using full optimization.

For the 37 plans for which a previous online-adapted plan was used for adaptation, the change in GTV-volume was (median [range]) 0.1 cm^3^ [− 1.1–12.0], whereas this was 0.0 cm^3^ [− 5.6–9.7] for the other 201 plans for which the reference plan was used.

Dose statistics for both GTV, PTV and the 2 cm ring around the PTV, are shown in Table 3. All values are displayed in percentage differences relative to the reference treatment plans. The difference in dose parameters for the full and weighted optimized plans is presented in Figure S2 of [Additional file 1].

**Table 3:** Relative differences (%) in dosimetric parameters of the GTV, PTV and 2 cm ring around the PTV (‘Ring’), between the adapted plans and the reference plans. All mean changes larger than 1% are marked in bold. GTV: Gross Tumor Volume, PTV: Planning Target Volume, SD: Standard Deviation

	ALL	Abdomen	Liver	Liver (non-SBRT)	Lung	Prostate	Prostate (non-SBRT)	Pelvis
**GTV**								
Dmean								
Median [range]	0.14 [−9.0–4.7]	−0.11 [−9.0–4.7]	0.23 [−2.5–3.4]	− 0.23 [− 0.98–0.56]	0.46 [− 1.9–4.6]	0.19 [− 1.2–2.3]	− 0.44 [− 1.1–1.3]	0.18 [− 2.5–1.5]
Mean ± SD	0.06 ± 1.5	− 0.26 ± 2.1	0.35 ± 1.4	− 0.17 ± 0.44	0.42 ± 1.3	0.40 ± 0.79	− 0.21 ± 0.70	0.00 ± 1.1
*P-value*	*0.04*	*0.56*	*0.21*	*0.31*	*0.06*	*0.005*	*0.16*	*0.61*
D95%								
Median [range]	− 0.11 [− 25.8–31.3]	−0.35 [− 25.8–31.3]	0.44 [− 8.7–5.8]	− 0.46 [− 1.2–0.78]	0.20 [− 2.2–5.4]	−0.03 [− 0.85–4.1]	−0.43 [− 1.2–1.3]	0.15 [− 1.7–1.5]
Mean ± SD	− 0.09 ± 4.5	− 0.68 ± 7.4	0.42 ± 2.7	−0.34 ± 0.64	0.41 ± 1.5	0.38 ± 1.2	−0.32 ± 0.66	0.09 ± 0.72
*P-value*	*0.71*	*0.008*	*0.14*	*0.10*	*0.21*	*0.27*	*0.07*	*0.46*
D98%								
Median [range]	−0.19 [− 23.7–44.7]	−0.57 [− 23.7–44.7]	0.53 [−9.3–10.8]	− 0.69 [− 2.7–0.92]	0.18 [− 2.1–5.4]	−0.04 [− 1.3–6.3]	−0.43 [− 1.2–1.1]	0.03 [− 2.4–1.6]
Mean ± SD	0.04 ± 5.3	−0.40 ± 8.6	0.47 ± 4.0	−0.61 ± 1.0	0.48 ± 1.5	0.63 ± 1.9	−0.30 ± 0.64	−0.03 ± 0.89
*P-value*	*0.34*	*0.002*	*0.15*	*0.08*	*0.11*	*0.39*	*0.08*	*0.97*
D2%								
Median [range]	0.46 [−11.6–4.4]	0.65 [− 11.6–4.4]	0.29 [− 3.2–2.7]	0.22 [− 0.42–1.0]	0.29 [− 2.7–2.9]	0.64 [− 1.5–4.2]	−0.43 [− 1.3–1.7]	0.47 [− 3.7–2.1]
Mean ± SD	0.40 ± 1.5	0.42 ± 2.0	0.36 ± 1.3	0.30 ± 0.41	0.37 ± 1.46	0.85 ± 1.0	− 0.02 ± 0.90	0.07 ± 1.4
*P-value*	*< 0.0001*	*0.007*	*0.26*	*0.03*	*0.10*	*< 0.0001*	*0.95*	*0.30*
**PTV**								
Dmean								
Median [range]	0.08 [−8.6–5.4]	− 0.11 [− 8.6–5.4]	0.23 [− 5.0–2.2]	−0.17 [− 1.3–0.23]	0.31 [− 2.4–3.2]	0.19 [− 1.2–1.3]	−0.17 [− 0.65–0.00]	0.24 [− 2.1–1.9]
Mean ± SD	0.01 ± 1.5	−0.29 ± 2.1	0.16 ± 1.6	−0.26 ± 0.57	0.40 ± 1.3	0.27 ± 0.46	−0.22 ± 0.25	0.09 ± 1.1
*P-value*	*0.02*	*0.54*	*0.13*	*0.12*	*0.05*	*< 0.0001*	*0.007*	*0.26*
D95%								
Median [range]	0.00 [− 26.4–13.4]	−0.26 [− 12.9–18.4]	0.18 [− 26.4–6.2]	− 0.34 [− 5.1–0.24]	−0.02 [− 2.0–4.4]	0.03 [− 0.86–1.2]	−0.05 [− 0.89–1.1]	0.36 [− 1.3–2.6]
Mean ± SD	−0.24 ± 3.6	−0.55 ± 5.2	−0.56 ± 5.8	**− 1.1** ± 1.7	0.08 ± 1.2	0.10 ± 0.44	− 0.05 ± 0.55	0.37 ± 0.96
*P-value*	*0.95*	*0.12*	*0.33*	*0.02*	*0.98*	*0.26*	*0.70*	*0.06*
D98%								
Median [range]	−0.04 [−31.6–25.4]	−0.19 [− 18.9–25.4]	−0.14 [− 31.6–24.4]	−0.33 [− 29.7–0.21]	−0.18[− 21.0–5.5]	0.18[− 2.4–5.9]	−0.16[− 2.0–1.4]	0.16[− 2.6–2.2]
Mean ± SD	−0.25 ± 5.4	−0.18 ± 5.7	0.37 ± 8.9	**−5.4** ± 10.8	−0.57 ± 3.6	0.74 ± 1.9	−0.15 ± 0.77	0.40 ± 1.17
*P-value*	*0.46*	*0.21*	*0.95*	*0.03*	*0.18*	*0.04*	*0.41*	*0.09*
D2%								
Median [range]	0.26[−43.6–3.9]	0.32[− 3.3–3.6]	0.25[− 43.6–3.1]	0.10[− 0.23–0.86]	0.38[− 2.5–3.9]	0.51[−1.6–3.7]	−0.29[− 1.4–0.96]	0.31[−3.7–2.1]
Mean ± SD	0.14 ± 3.1	0.37 ± 1.4	**−1.3** ± 8.7	0.15 ± 0.30	0.41 ± 1.5	0.62 ± 0.91	− 0.10 ± 0.67	−0.11 ± 1.5
*P-value*	*< 0.0001*	*0.04*	*0.21*	*0.17*	*0.15*	*< 0.0001*	*0.80*	*0.71*
**Ring**								
Dmean								
Median [range]	0.80[−15.8–13.1]	1.0[−7.2–8.4]	1.6[− 4.5–8.1]	−0.28[− 4.7–2.7]	0.80[−4.2–7.8]	− 0.17[− 15.8–6.8]	0.00[− 10.0–2.7]	1.5[− 1.9–13.1]
Mean ± SD	**1.0** ± 3.6	**1.4** ± 3.2	**1.6** ± 3.4	−0.14 ± 2.2	**1.8** ± 2.9	−0.89 ± 5.3	−0.51 ± 2.7	**2.5** ± 3.4
*P-value*	*< 0.0001*	*0.0002*	*0.05*	*0.89*	*0.01*	*0.89*	*0.85*	*0.001*
D5%								
Median [range]	0.56 [−10.5–6.8]	0.72[− 4.3–6.0]	0.21[− 2.0–6.8]	0.00[−1.7–0.86]	1.6[−3.5–4.9]	− 0.10[− 10.5–3.0]	0.34[− 1.1–1.9]	1.3[− 0.24–5.8]
Mean ± SD	0.64 ± 2.1	0.71 ± 1.5	0.90 ± 2.1	− 0.07 ± 0.79	**1.3** ± 2.0	−0.70 ± 3.3	0.35 ± 0.78	**1.6** ± 1.4
*P-value*	*< 0.0001*	*< 0.0001*	*0.11*	*0.84*	*0.002*	*0.86*	*0.11*	*< 0.0001*

**Fig. 3:**
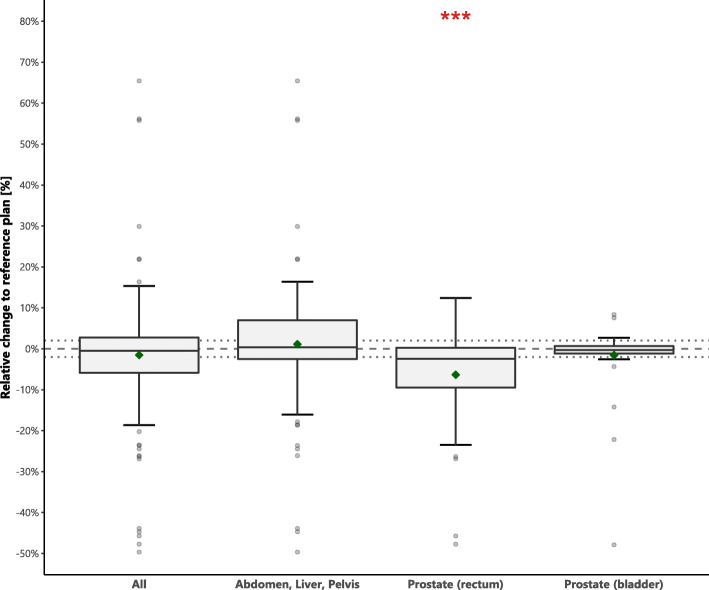
Boxplot indicating relative change of OAR D_1.0cc_ of adapted plans with respect to reference treatment plans. For abdomen, liver and pelvis, either stomach, bowel or duodenum was evaluated. For prostate, both rectum and bladder were evaluated. Diamonds indicate the mean. Dashed lines indicate 0% and dotted lines indicate ±2% differences. Asterisks indicate significance: *p*-value< 0.05 (*), *p*-value< 0.01 (**) and *p*-value< 0.001 (***)

Figure 3 shows the results (D_1.0cc_) for the OARs. The last column of Table 1 indicates the OARs that were considered for abdomen, liver, pelvis, and prostate. Note that not for all patients a serial OAR was present close to the tumor.

The differences between the average dose parameters of all online-adapted treatment plans per patient and the reference treatment plans are shown in Figure S3 and S4 of [Additional file 1].

## Discussion

In MRgART, treatment plans are adapted to account for inter-fractional changes in patient anatomy using MRI scans that are acquired while the patient is in treatment position. The time for the adaptation process should preferably be as short as possible, while maintaining high treatment plan quality. Therefore, in this study, the (dosimetric) plan quality of online-adapted treatment plans was compared to that of the reference treatment plans, which were considered optimal for the purpose of this study.

A significant increase in volume from the reference to the online-adapted plan was observed in both GTV and PTV, which could potentially be explained by radiotherapy induced edema or tumor swelling. A change in GTV or PTV volume might also be due to time pressure during adaptation, although it is yet unknown if time pressure could have caused a systematic increase.

A previous study reported an average increase in GTV and PTV of 0.5 cm^3^ and 0.8 cm^3^, respectively, for 25 peripherally located lung tumors [15]. This study mentioned potential uncertainty in contours caused by inter-observer variability and lack of experience in MR-based contouring of small lung tumors. We observed smaller changes: absolute mean decrease of 0.07 cm^3^ in GTV and 0.6 cm^3^ increase in PTV, due to the initialization of the local rule to not adapt the GTV in the lungs (lung tumors in this study were peripherally located). Palacios et al. reported a large spread in GTV changes for the abdominal region, which were caused by rotations and deformations, potentially due to tumor progression [23]. In that study, PTV volume was largely reduced (15%) in two patients to achieve further OAR sparing.

The subgroup analysis does not show a clear difference in GTV-volume changes between online-adapted plans based on previously online-adapted plans (*n* = 37) or the reference plans (*n* = 201). But, this change of workflow within the study timeframe is a limitation. Note that for all analyses in this study, the online-adapted plans were compared to the reference plans.

The target coverage was comparable between the online-adapted plans and the reference plans. The median change in both GTV and PTV dose parameters stayed within 1%. The same is the case for the GTV-D_mean_, which is considered an important parameter for local tumor control. Small changes compared to the reference plan were observed in the online-adapted plans; meaning that it was possible to maintain a high effective dose in the online-adaptive workflow [24, 25]. Nevertheless, some changes in PTV- and GTV dose parameters may seem larger than expected in treatments on the conventional linac. Because of the possibility to perform daily adaptations, the dose prescribed to the PTV is larger than treatment on the conventional linac, whereas the same OAR constraints were used. In each adaption, the aim was to obtain the best coverage of PTV and GTV without exceeding the tolerance limits for OARs. Overall, the largest range of changes in dose parameters was observed for abdominal patients. For this group, OAR constraints were often violated because one of the organs was in close proximity to the PTV. Also, there is a lot of movements of close organs caused by digestion. Therefore, often the PTV had to be compromised, and most plans required full optimization. The amount of overlap between the OAR and PTV, as well as how much the compromise on the PTV-D95% is, depends on the PTV margin.

In case of overlapping organs, help structures (e.g. GTV_PH and PTV_PH) might have been created that are cropped away from the OAR, which were then used for treatment planning to ensure meeting the OAR dose tolerances. This is according to the concept of simultaneous integrated protection [26]. For patients with varying overlap between OARs and PTV, this can lead to large changes in the PTV or GTV coverage between the reference plans and daily-adapted plans. For instance, the outliers in the abdomen patients can be attributed to one patient with a kidney tumor. The stomach overlapped with the GTV and PTV in the reference plan, so GTV_PH and PTV_PH were used. In two fractions, the stomach did no longer overlap with the target and thus the PTV-D_98%_ increased by 25% in both fractions, the GTV-D_95%_ increased by 29 and 31%, and the GTV-D_98%_ increased by 41 and 45%. But since GTV_PH and PTV_PH were used to optimize the treatment plans, the dose parameters in these structures remained stable (within 2%), indicating a robust behavior of the optimizer. Similarly, the average significant decrease in PTV-D_98%_ for the liver cases was mainly caused by one patient for which the stomach overlapped more with the PTV in two online-adapted plans than in the reference plan, causing decreases in the PTV coverage with respect to the reference plan of more than 25%. In general for the liver (non-SBRT) patients, who all had large, diffuse tumors, also the largest GTV and PTV volume changes were observed. These large anatomical changes (e.g. changes in GTV volume) make it more difficult to compare the treatment plan quality of online-adapted plans with respect to reference treatment plans.

Dose spillage (e.g. dose in the 2 cm ring) was slightly increased for all plans. This might be caused by the limited time that is available for optimizing the plans. Significant dose spillage increase was observed for both optimization strategies. For some patients an OAR was often overlapping or in direct proximity to the PTV, so daily adaptations of the contours and full optimization were required. This was the case for a pelvic patient, for which a 13.1% increase in ring D_mean_ was observed. In general, OAR dose (D_1.0cc_) was comparable between the online-adapted plans and the reference plans, and slightly decreased for the rectum. A limitation of this study is that OAR-D_mean_ could not be assess to further evaluate OAR dose, since optimization is only performed in the MRI image-slices containing the 2 cm ring.

The treatment time was not recorded for all patients included in this study, which is a limitation of this retrospective study. Using the available data, patients were found to be on the treatment table on average for 65 min, with an average adaptation time of 24 min. Adaptive treatment time could be reduced by shortening the time required for contour adaptations, which could be achieved by improved deformable registrations, or automatic segmentation using artificial intelligence [27]. Further reduction in appointment time could be achieved by making better-informed decisions about the time spent on adapting and optimizing the treatment plan. Previous studies have shown that for different tumor sites that the majority of fractions the optimized plan was improved compared to the predicted plan, but that online adaption might not always be beneficial [12, 14, 15, 17, 18, 23, 28]. Our study did not investigate this aspect, and neither were the improvements of a full optimization over a weight optimization studied. The optimization strategy is currently subject to high inter-observer variability, which is a limitation of this study. Further studies are required to investigate the benefits of a full optimization; to provide guidelines for the choice of either the predicted, weighted optimized or fully optimized plan, which could potentially reduce time on the treatment table for some patients. The current study only shows that for small changes in GTV (< 2%), a smaller fraction of treatment plans was optimized with full optimization, indicating that the amount of GTV volume changes might be a useful indicator for the best suitable optimization approach, considering the trade-off between treatment time and treatment plan quality [28]. In the future, automated planning could possibly contribute to the reduction of treatment time, while improving overall plan quality and reducing inter-planner variability [29].

## Conclusions

This study demonstrates the feasibility of MR-guided online-adaptive radiotherapy, and shows a comparable dosimetric plan quality in online-adapted treatment plans to those achieved in the reference treatment plan. The results were different for each treatment site, indicating that online treatment plan evaluation should have site-specific protocols.

## Supplementary information

**Additional file 1.**

## Data Availability

All data generated or analyzed during the current study are available from the corresponding author on reasonable request.

## Abbreviations

bSSFP: Balanced steady-state free precession
CBCT: Cone-beam computed tomography
CT: Computed tomography
DVH: Dose-volume histogram
FFF: Flattening filter-free
GTV: Gross tumor volume
IGRT: Image-guided radiotherapy
IMRT: Intensity-modulated radiotherapy
Linac: Linear accelerator
MLC: Multi-leaf collimator
MRgART: Magnetic resonance guided adaptive radiotherapy
MRI: Magnetic resonance imaging
OAR: Organ at risk
PTV: Planning target volume
SAD: Source-to-axis distance
SBRT: Stereotactic body radiotherapy
SD: Standard deviation
TPS: Treatment planning system
