# Supplementary material for: Treatment plan quality during online adaptive re-planning
Source: Radiat Oncol. 2020 Aug 21;15:203. doi: 10.1186/s13014-020-01641-0 (PMC7441614; doi:10.1186/s13014-020-01641-0)
Supplement: Supplementary file 1 — Additional file 1. [file 13014_2020_1641_MOESM1_ESM.pdf]

Supplementary Material

Treatment plan quality during online adaptive re-planning

Janita van Timmeren, Madalyne Chamberlain, Jérôme Krayenbuehl, Lotte Wilke, Stefanie Ehrbar, Marta Bogowicz, Mariangela Zamburlini, Nicolaus Andratschke, Helena Garcia, Matea Pavic, Panagiotis Balermpas, Chaehee Ryu, Matthias Guckenberger, Stephanie Tanadini-Lang

1. **PTV changes**
2. **Weighted versus full optimization**
3. **Average differences per patient**

1. PTV changes

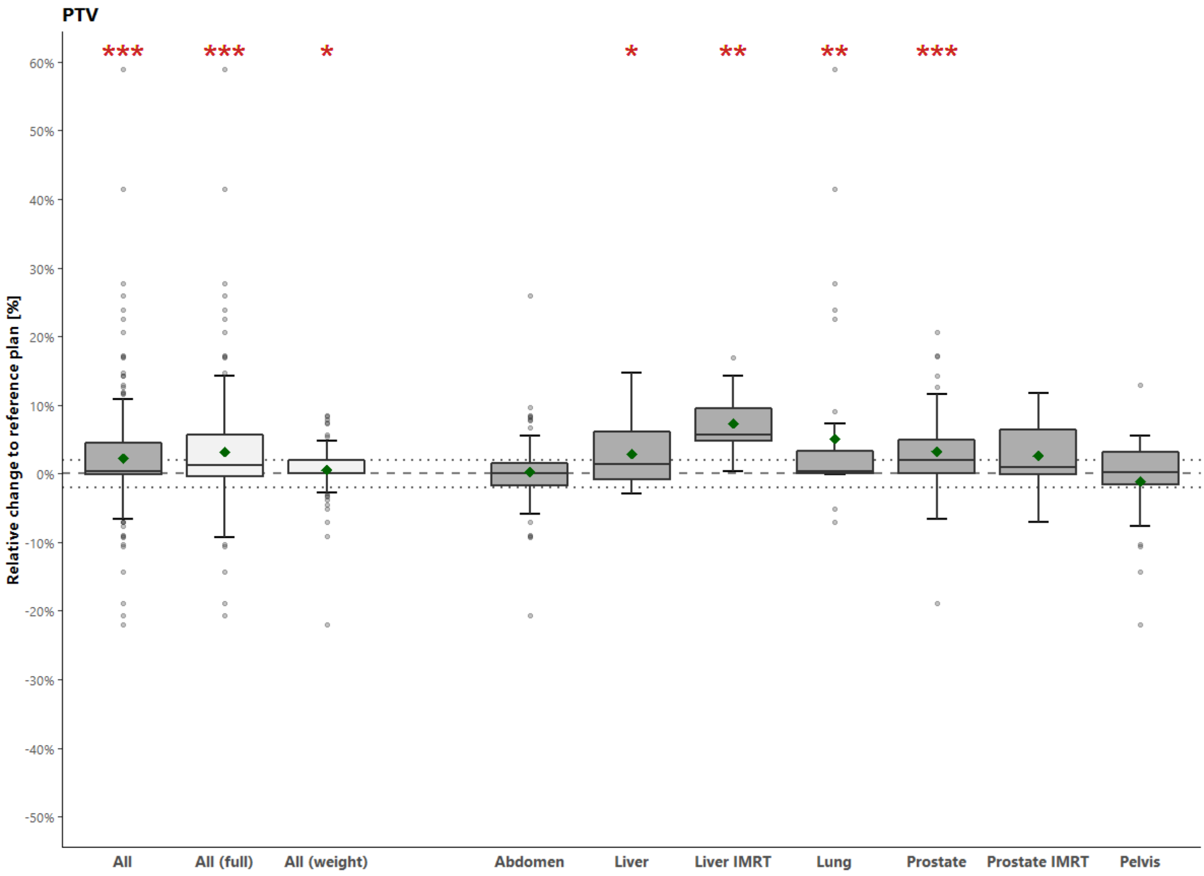

Figure S1: Boxplots indicating the relative difference in PTV of the adapted plans compared to the respective reference treatment plans. Diamonds indicate the mean. Dashed lines indicate 0% and dotted lines indicate ±2% differences. Asterisks indicate significance: p-value<0.05 (*), p-value<0.01 (**) and p-value<0.001 (***).

2. Weighted versus full optimization

In this analysis, the difference in dose parameters between the online-adapted treatment plans and the reference plans were calculated for those plans that were optimized with the full optimization or weight optimization strategy. Note that all online-adapted plans were pooled together; the analysis was not performed per treatment site due to low patient numbers in each subgroup.

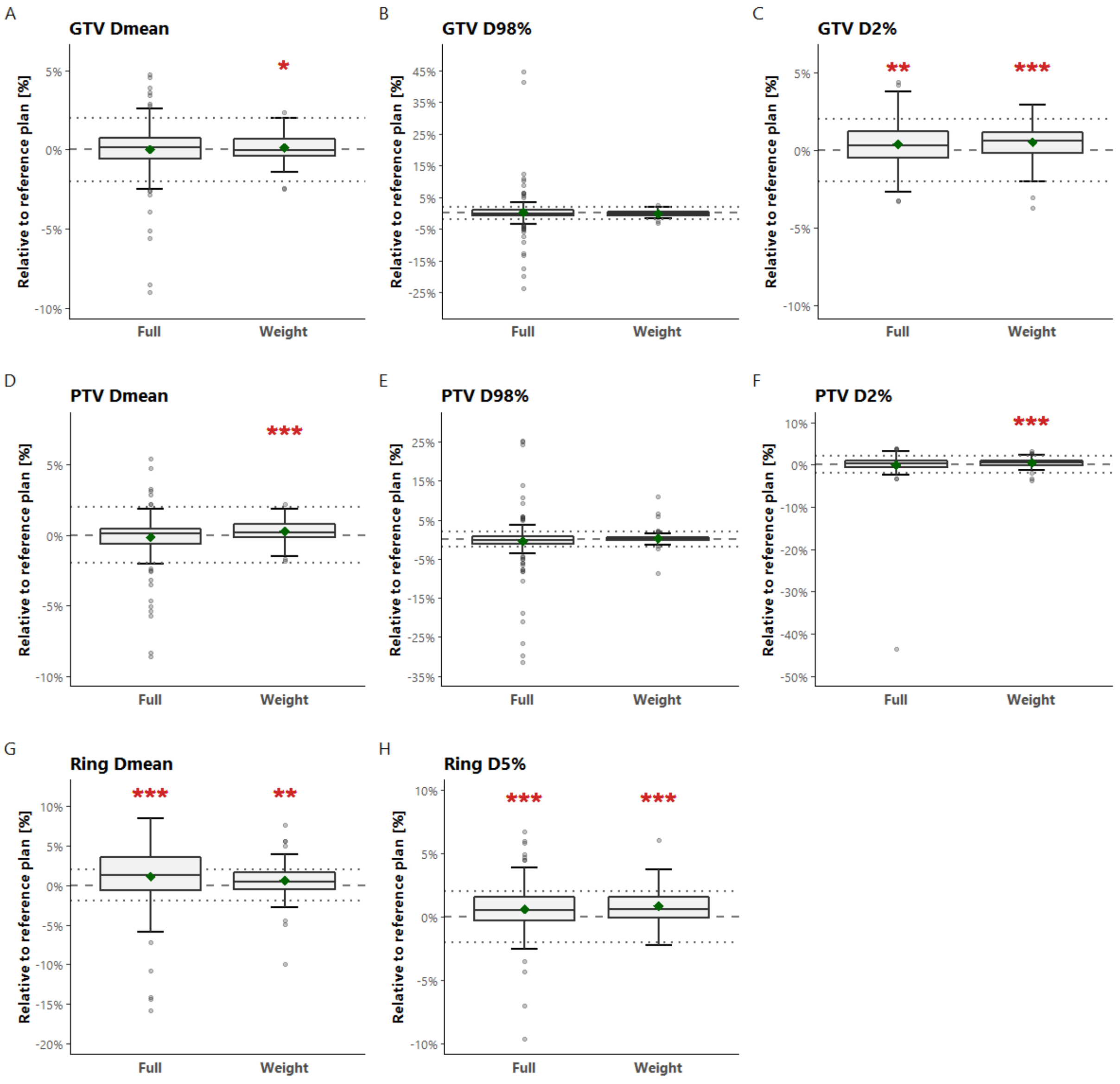

Figure S2: Boxplots representing the percentage differences in dose statistics with respect to reference treatment plans, only for those adapted plans that required a full optimization (left) or only weight optimization (right). (A-C) GTV, (D-F) PTV, (G-H) 2 cm ring around the PTV. Dashed lines indicate 0% and dotted lines indicate ±2% differences. Asterisks indicate significance: p-value<0.05 (*), p-value<0.01 (**) and p-value<0.001 (***). GTV: Gross Tumor Volume, PTV: Planning Target Volume.

3. Average differences per patient

Figure S3 shows the mean difference per patient of the online-adapted treatment plans with respect to the reference treatment plan.

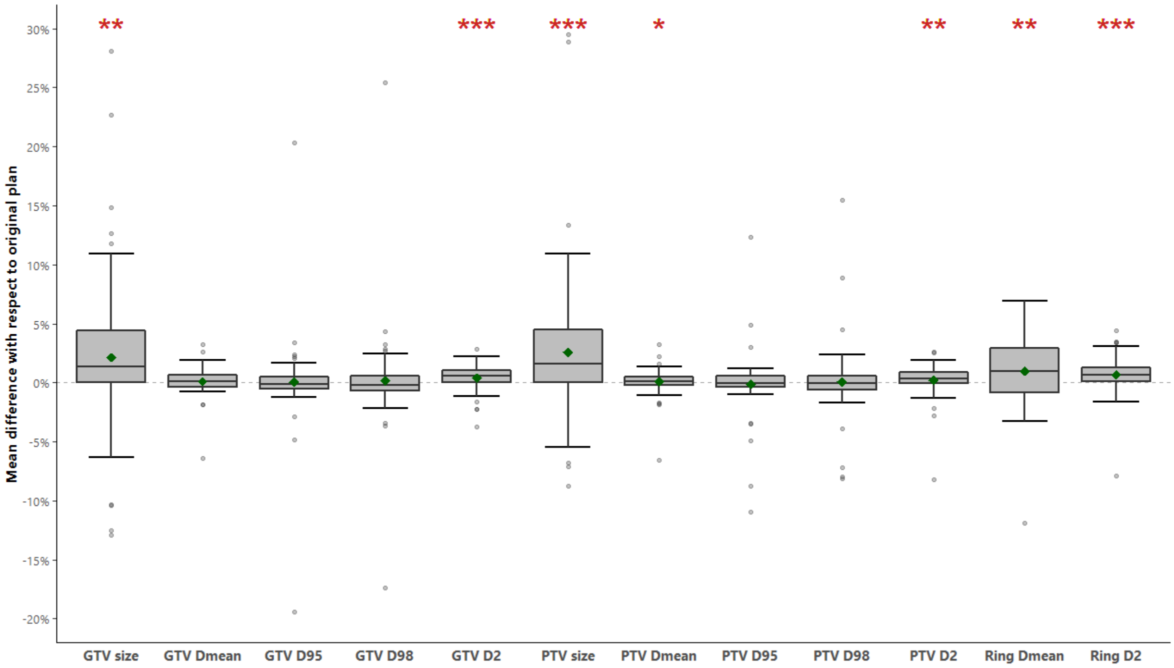

Figure S3: Boxplots indicating the average difference of adapted plans per patient with respect to original treatment plans. Diamonds indicate the mean. Dashed lines indicate 0% and dotted lines indicate ±2% differences. Asterisks indicate significance: p-value<0.05 (*), p-value<0.01 (**) and p-value<0.001 (***).

Figure S4 shows for the GTV dose parameters ($D_{95\%}$ is omitted here, because it is comparable to the $D_{98\%}$), the average change from the reference treatment plan, sorted from largest increase to largest decrease. The colors indicate the different treatment sites.

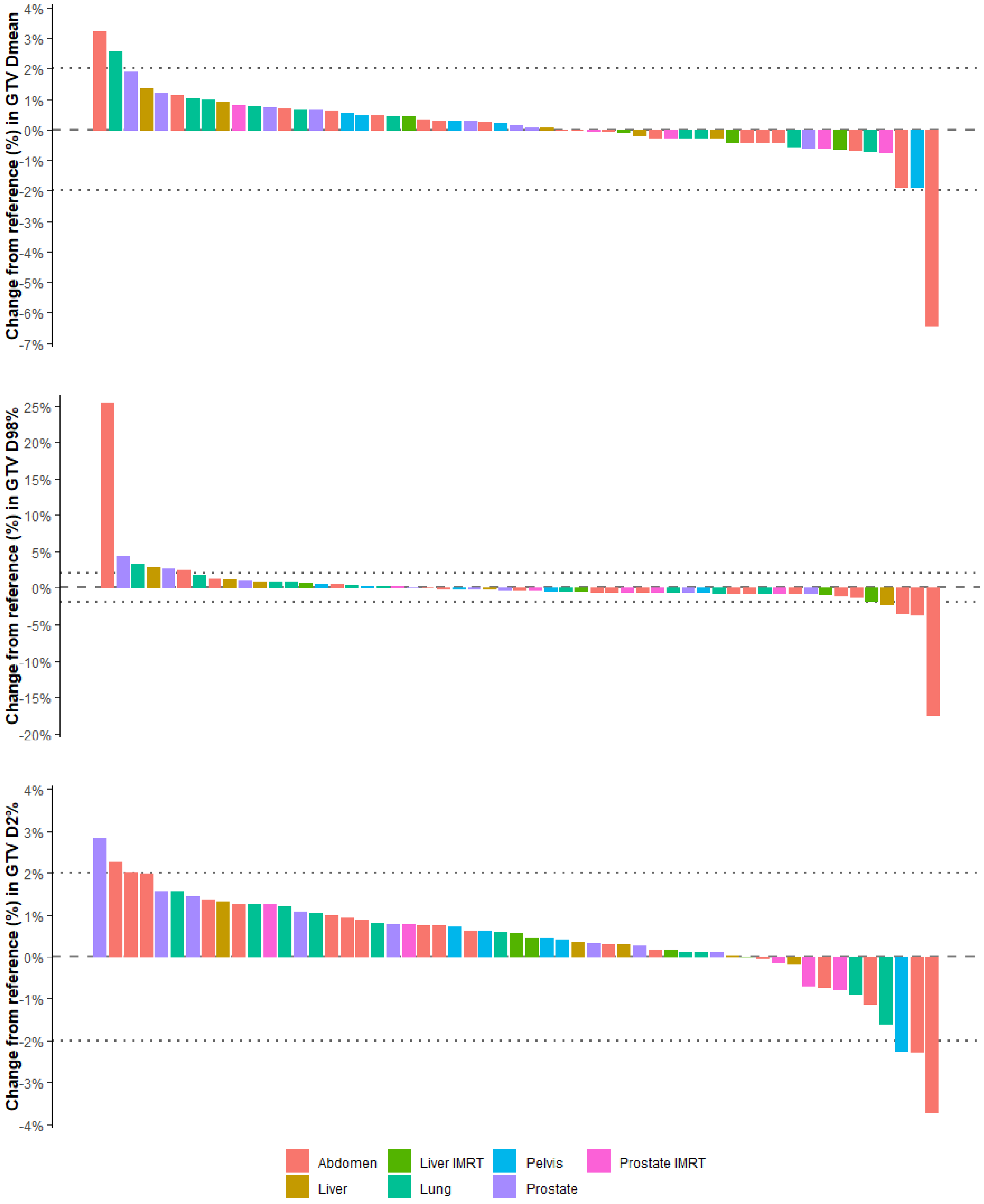

Figure S4: Waterfall plots for GTV parameters D_{mean}, $D_{98\%}$ and $D_{2\%}$, where each bar represents one patient, and is the average difference of all online-adapted plan of that patient and the reference plan. Dashed lines indicate 0% and dotted lines indicate ±2% differences.
